# Correction: Expanding analytical tools for characterizing ultrasmall silica-based nanoparticles

**DOI:** 10.1039/c8ra90077a

**Published:** 2018-10-04

**Authors:** B. Yoo, K. Ma, U. Wiesner, M. Bradbury

**Affiliations:** Department of Radiology, Sloan Kettering Institute for Cancer Research New York New York 10065 USA yoob@mskcc.org; Department of Chemistry, Hunter College New York New York 10065 USA; Department of Materials Science & Engineering, Cornell University Ithaca New York 14853 USA ubw1@cornell.edu; Molecular Pharmacology Program, Sloan Kettering Institute for Cancer Research New York NY 10065 USA

## Abstract

Correction for ‘Expanding analytical tools for characterizing ultrasmall silica-based nanoparticles’ by B. Yoo *et al.*, *RSC Adv.*, 2017, **7**, 16861–16865.

The authors regret that a Conflicts of interest section was not shown in the original article. The correct Conflicts of interest section is shown below.

## Conflicts of interest

The authors declare the following competing interests: the subject matter presented herein may be covered by one or more US or international patent applications. MSK, Cornell, and two investigators involved in this study have a financial interest in Elucida Oncology, Inc.

The Royal Society of Chemistry apologises for these errors and any consequent inconvenience to authors and readers.

## Supplementary Material

